# Molecular Detection and Phylogenetic Analysis of Deformed Wing Virus and Sacbrood Virus Isolated from Pollen

**DOI:** 10.3390/vetsci10020140

**Published:** 2023-02-10

**Authors:** Ralitsa Balkanska, Rositsa Shumkova, Nedyalka Atsenova, Delka Salkova, Heliana Dundarova, Georgi Radoslavov, Peter Hristov

**Affiliations:** 1 Department “Special Branches”, Institute of Animal Science, Agricultural Academy, 2230 Kostinbrod, Bulgaria; 2 Research Centre of Stockbreeding and Agriculture, Agricultural Academy, 4700 Smolyan, Bulgaria; 3 Department of Animal Diversity and Resources, Institute of Biodiversity and Ecosystem Research, Bulgarian Academy of Sciences, 1113 Sofia, Bulgaria; 4 Department of Experimental Parasitology, Institute of Experimental Morphology, Pathology and Anthropology with Museum, Bulgarian Academy of Sciences, 1113 Sofia, Bulgaria; 5 Department of Ecosystem Research, Environmental Risk Assessment and Conservation Biology, Institute of Biodiversity and Ecosystem Research, Bulgarian Academy of Sciences, 1113 Sofia, Bulgaria

**Keywords:** honey bee viruses, RT-PCR, pollen, phylogeny, epidemiology

## Abstract

**Simple Summary:**

Honey bee viruses annually cause serious damage to beekeeping worldwide. Therefore, it is important to accumulate more data regarding routes by which viruses spread. This study was carried out to investigate six of the most widely distributed honey bee viruses in pollen samples either collected from different regions in Bulgaria or purchased directly from the trade market. The obtained results showed the presence of deformed wing virus and sacbrood virus with different frequencies within the examined regions. The phylogenetic analyses of Bulgarian isolates were performed considering the most similar worldwide strains available in the GenBank database. Consequently, it has been concluded that pollen is a valuable source for the detection of honey bee viruses, which provides additional information regarding the horizontal way of spreading of these viruses.

**Abstract:**

Among many pathogens and pests, honey bee viruses are known as one of the most common cause of diseases in honey bee colonies. In this study, we demonstrate that pollen grains and bee bread are potential sources of viral DNA. We extracted DNA from 3 types of pollen samples: directly provided by beekeepers (*n* = 12), purchased from trade markets (*n* = 5), and obtained from honeycombs (bee bread, *n* = 10). The extracted DNA was used for molecular detection (RT-PCR analysis) of six of the most widely distributed honey bee viruses: deformed wing virus, sacbrood virus, acute bee paralysis virus, black queen cell virus, Kashmir bee virus, Israeli acute paralysis virus, and chronic bee paralysis virus. We successfully managed to establish only the deformed wing virus (DWV) and the sacbrood virus (SBV), with different distribution frequencies depending on the territory of the country. The phylogenetic analyses of Bulgarian isolates were performed with the most similar sequences available in molecular databases from other countries. Phylogenies of Bulgarian viral strains demonstrated genetically heterogeneous populations of DWV and relatively homogenous populations of SBV. In conclusion, the results obtained from the current study have shown that pollen is a valuable source for molecular detection of honey bee pathogens. This allows epidemiological monitoring of honey bee diseases at a regional and a national level.

## 1. Introduction

Western honey bees (*Apis mellifera* L.) are the world’s most dominant pollinators. As pollinator generalists, honey bees are of great importance to humans and to agricultural and natural ecosystems [1,2,3]. They contribute to pollination of agricultural crops, wild flora, and natural vegetation worldwide [4,5]. Furthermore, honey bees play a key role as providers of pollination services to many endemic plant species, thereby preserving these species from extinction and maintaining plant diversity in many ecosystems [6,7].

Among many pests and pathogens, honey bee viruses represent one of the most serious threats to honey bee health and survival [8,9,10,11]. These hidden, small biological agents are particularly dangerous because the diseases they cause occur without a clear clinical picture or pathognomonic symptoms [12,13,14]. Until now, over 25 honey bee-associated viruses have been detected [15,16]. A large number of studies have shown the geographical distribution of deformed wing virus (DWV), sacbrood virus (SBV), acute bee paralysis virus (ABPV), black queen cell virus (BQCV), the Kashmir bee virus (KBV), the Israeli acute paralysis virus (IAPV), and the chronic bee paralysis virus (CBPV) [17,18,19,20]. The viral genome of these viruses usually consists of a positive-strand RNA which codes structural (capsid) and nonstructural viral proteins, and which belongs to two major families of Dicistroviridae and Iflaviridae (part of the group within the order Picornavirales) [21,22,23]. The viral genome of a member of the genus Iflavirus (DWV and SBV) has a size ranging from 8800 to 10,100 bp and contains a single large open reading frame (monopartite genome), which includes gene encoding for capsid proteins at the 5′ end and gene encoding for nonstructural proteins at the 3′ end of the genome [24,25]. Unlike the members of the genus Iflavirus, those of the genus Dicistovirus (ABPV, BQCV, KBV, and IAPV) have a bicistronic RNA genome of approximately 8000–10,000 bp, which contains two main non-overlapping open reading frames (ORFs) [26,27]. The positive single-stranded RNA (+ssRNA) is organized as follows: a 5′ untranslated region (UTR), followed by two open reading frames (ORF 1 and ORF 2), separated by an intergenic region (IGR). ORF 1 encodes the nonstructural proteins, while ORF 2 encodes the structural proteins [28,29]. The only unclassified virus so far is the CBPV [30]. The genome organization of this positive single-stranded RNA virus revealed the presence of two major RNA fragments (bipartite virus) [31]. Seven overlapping open reading ORFs were identified, three on RNA 1 and four on RNA 2. Only ORF 3 on RNA 1 encoded nonstructural proteins, while ORFs on RNA 2 encoded structural proteins [31]. By means of deep sequencing analysis, the presence of a novel honey bee virus was found—namely, the Lake Sinai Virus strain 1 and 2 (LSV 1 and 2). Both LSV genomes display similarities to the RNA 1 molecule of the chronic bee paralysis virus (CBPV) [32]. The gene arrangement of ORF 1 in LSV 1 and 2, as well as CBPV, encode an RNA-dependent RNA polymerase (RdRp) in a similar way.

Forager bees are typically used as target biological objects for the molecular detection of honey bee viruses [33,34]. Alternatively, viruses can be detected by molecular analysis of some bee products, the most suitable being honey and pollen [35,36,37]. This approach has a number of advantages over the conventional one. There is no need to capture and examine foragers in the first place. Second, honey and pollen samples can be derived from more than one colony or beehive and even from more than one apiary. Usually, honey and pollen are mixed and can be commercially obtained and/or directly provided by beekeepers [38]. This determines the third advantage of this approach, i.e., monitoring of a large number of pathogens and pests of bee colonies over a certain period of time at a regional, national, and even global level.

The presence of honey bee viruses in pollen and honey poses a risk of infection of healthy bee colonies, either by frames from infected colonies or by feeding with honey and/or pollen (bee bread or pollen grains) [39]. Alternatively, healthy honey may be infected by feeding with infected honey and pollen when the food reserves in the bee colony are running out and additional feeding is required. There are still insufficient data on the transmission of viruses through bee products. Considering that this horizontal way of spreading of viruses hides the danger of introducing new viruses, the emergence of new strains of an already existing virus through recombinant processes should not be overlooked [39].

The aim of this study was to determine whether pollen (pollen grains and/or bee bread) is a potential source of honey bee viruses. We focused on six of the most widespread viruses worldwide—DWV, BQCV, SBV, ABPV, CBPV, and KBV. We also performed a phylogenetic analysis to reveal and compare the nucleotide sequences of the Bulgarian isolates with different similar isolates worldwide, as well as to assess the genetic relationship between Bulgarian strains of various geographic origins.

## 2. Materials and Methods

### 2.1. Study Area and Sample Collection

**Figure 1:**
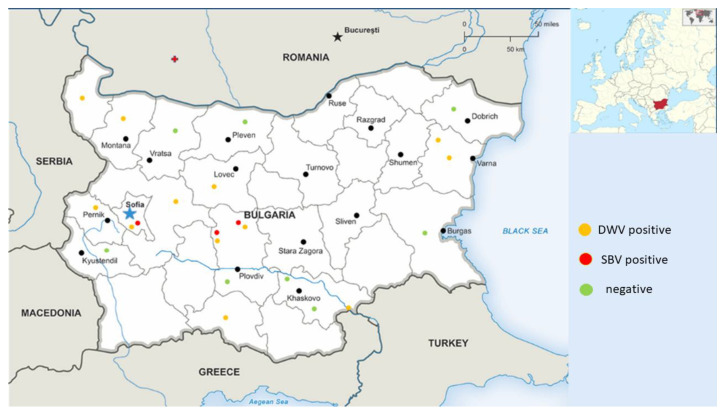
Map showing the geographic localization and the positive and negative (DWV and SBV) pollen samples.

A total of 29 pollen samples were collected in 2021 and 2022 from different regions of Bulgaria (Figure 1). These samples were directly provided by beekeepers (*n* = 12), purchased from trade markets (*n* = 5), or obtained from honeycombs (*n* = 12, bee bread). All samples (except bee bread) were not from a single hive, as they were derived by the routine procedures for preparations. An efficient, humane, and safe way to collect pollen is with the help of special devices (usually pollen traps) that are placed at the entrance of the hive. Briefly, foragers carrying pollen are forced to pass through the pollen trap, which is a grid with precisely calibrated holes. Some of the pollen grains are blown off their feet and fall through the net into drawer-like boxes below. In this way, the bees are not traumatized nor physically damaged, and the method ensures that there is enough pollen left for the needs of the bees. Pollen is collected daily in humid weather and less frequently in dry weather. In order to avoid deterioration of its quality and the appearance of bacteria and molds, the harvested pollen is dried in special dryers, or simply in a warm and dry place, at a temperature no higher than 40 °C.

When selecting the samples, we attempted to cover different regions of the country in order to obtain information regarding the health status of bee colonies and the epizootic situation in the country.

### 2.2. Total RNA Extraction and Copy DNA (cDNA) Synthesis

Before RNA isolation, 100 mg of all obtained pollen samples were homogenized with sterile diethylpyrocarbonate (DEPC)-treated water (to prevent the action of RNases) using a ceramic mortar and pestle. After 2 centrifugations at 10,000× *g* for 1 min, the supernatant was used for RNA extraction. Total RNA isolation was performed using a GeneMATRIX Universal RNA Purification Kit (Cat. No. E3598, EURx Ltd., Gdansk, Poland). The quality and quantity of the purified RNA were evaluated spectrophotometrically and by a 2% native agarose gel electrophoresis. Copy DNA (cDNA) synthesis was performed using a smART First Strand cDNA Synthesis Kit (Cat. No. E0804, EURx Ltd., Gdansk, Poland) according to the manufacturer’s instructions. The reaction mixture contained 14 µL of purified RNA, 4 µL of 5× NG cDNA Buffer, 1 µL NG dART RT Mix, and 1 µL random hexamer primers in a total volume of 20 µL. Then, the mixture was incubated at 25 °C for 10 min, followed by 60 min at 50 °C, and finally, the reactions were terminated by incubating at 85 °C for 5 min. The cDNA was stored at—20 °C prior to analysis.

### 2.3. Gene Selection and RT-PCR Amplification

Gene selection was carried out on the basis of viral sequences available in the GenBank database (https://www.ncbi.nlm.nih.gov/genbank/, accessed on 14 December 2022). Most viral sequences are based on the amplification of fragments of genes for structural proteins (VP); sequences from nonstructural proteins (mainly RNA-dependent RNA polymerase) are less commonly observed, and full genome sequences of honey bee viruses are also present. The parts of the viral genome sequences described above were chosen for analysis based on the availability of most sequences of other countries’ viral isolates. We used the primers designed by Stoltz et al. [40] for DWV (which amplified a part of DWV nonstructural protein genes), covering nucleotide positions of the viral genome 8556–8960 bp (RefSeq Acc. No. NC_004830) [41]; Tentcheva et al. [42] for SBV (amplified the part of nonstructural protein genes, nucleotide position 7.747–8.172 bp) (RefSeq Acc. No. NC_002066) [43], ABPV (amplified the part of capsid protein gene, nucleotide position 5.270–5.721 bp) (RefSeq Acc. No. NC_002548) [44] and BQCV (amplified a part of the orf 1 gene, i.e., nonstructural proteins, nucleotide position 4.611–5.034 bp) (RefSeq Acc. No. NC_002066) [45]; Ribière et al. [46] for CBPV (amplified the part of the orf 3 gene, i.e., RNA-dependent RNA polymerase nucleotide position 1–455 bp) (RefSeq Acc. No. NC_010711) [30]; and Kashmir bee virus (amplified the part of nonstructural polyprotein gene nucleotide position 5406–5804 bp) (RefSeq Acc. No. NC_004807) [47]. All primer sets used for RT-PCR amplification are shown in Table 1.

All RT-PCR mixtures contained 25 µL of Color Taq PCR Master Mix (2×) (Cat. No. E2525, EURx Ltd., Gdansk, Poland), 1 µM of each virus-specific primer (FOR/REV), and 5 µL of template cDNA for a total volume of 50 µL. All PCR amplifications were carried out using a LifeExpress Classic Thermal Cycler (BIOER Technology Co., Ltd., Kampenhout, Belgium) under the following conditions: initial denaturation at 94 °C for 5 min; 35 cycles (denaturation at 94 °C for 30 s; primer annealing at 56 °C for 30 s; extension at 72 °C for 30 s), and final extension at 72 °C for 10 min. Negative and positive controls were included in each run of the RT-PCR reaction. A negative control of the RT-PCR reactions was established through incubating the samples in the absence of reverse transcriptase enzymes, or using cDNA template that was generated using random-hexamer primers and including the primer set for each virus in the RT-PCR. The RNA samples that were extracted from honey bees and confirmed to be virus-positive in a previous RT-PCR assay of the research team served as positive controls [48]. The RT-PCR products were visualized on 2% agarose gel stained with SimplySafe™ (Cat. No. E4600, EURx Ltd., Gdansk, Poland). The fragment size was determined using MassRuler Low Range DNA Ladder (Cat. No. SM0383, Thermo Fisher Scientific Inc., Waltham, MA, USA). The successfully amplified products were purified by a PCR.

A purification kit (PCR/DNA Clean-Up Purification Kit, Cat. No. E3520, EURx Ltd., Gdansk, Poland) was used and sequenced in both directions by a PlateSeq kit (Eurofins Genomics, Ebersberg, Germany).

### 2.4. Bioinformatics and Phylogenetic Analysis

All obtained DNA sequences were manually edited and aligned using the MEGA v. 11 software [49]. The complete viral genomes DWV (Acc. No. NC_004830) [41] and SBV (Acc. No. NC_002066) [43] were used as reference sequences. The obtained sequences, DWV (382 bp) and SBV (417 bp), were deposited in the GenBank database, National Biotechnology Information Center (NCBI), under accession numbers OP821136–OP821143 and OP821144–OP821145, respectively. The obtained sequences (DWV and SBV) covered part of the 5′ end of the region of the viral genome (structural proteins). Searching the GenBank database revealed the most similar sequences for both structural and nonstructural proteins; therefore, these sequences were included in the analysis. The honey bee viruses (DWV, Acc. No. MG599458–MG599464; SBV, Acc. No. MG649495–MG649499) isolated from honey bees as a source of biological material [48] were also included in the phylogenetic analysis in order to compare the possible similarities and/or differences between isolates from honey bees and pollen. After extracting appropriate sequences from GenBank, all sequences were aligned using the MUSCLE algorithm [50], and then the best-fit substitution model was selected for the purpose of constructing each viral phylogeny. Evolutionary analyses were conducted in MEGA11 [49]. The evolutionary history was inferred using the neighbor-joining method [51]. The percentage of replicate trees in which the associated taxa clustered together in the bootstrap test (10,000 replicates in each case) is shown above the branches [52]. The evolutionary distances were computed using the maximum composite likelihood method [53] and are shown in units of the number of base substitutions per site. All positions containing gaps and missing data were eliminated (complete deletion option).

## 3. Results

### 3.1. Distribution of Detected Honey Bee Viruses in Different Regions of the Country

**Figure 2:**
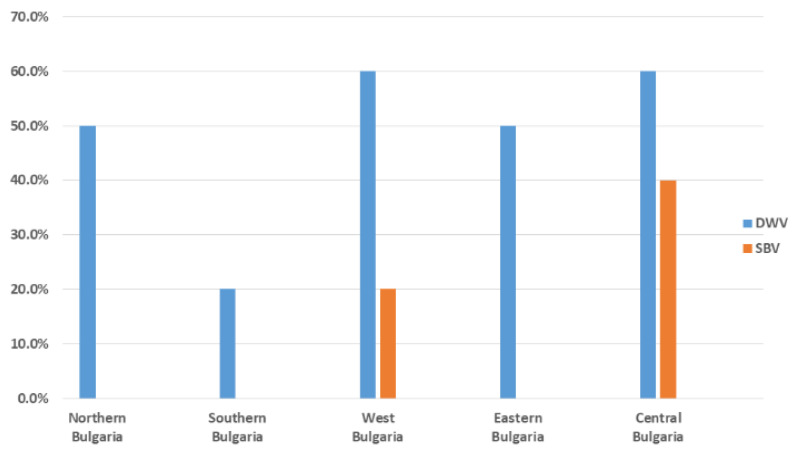
Frequency (%) of the positive (successfully amplified fragment) pollen samples (DWV and SBV) in different regions of the country. The analysis included a total of 27 pollen samples: directly provided by beekeepers (*n* = 12), purchased from the trade markets (*n* = 5), or obtained from honeycombs (bee bread, *n* = 10).

Pollen samples were collected from a total of 14 different regions in Bulgaria in order to provide more comprehensive information about the epizootic situation (Figure 1). We were able to detect only DWV and SBV in each of the three studied pollen types (from trade markets, directly provided by beekeepers, and bee bread). DWV was observed with the highest frequency among all samples (12/25, 48%). SBV was found with a significantly lower frequency (3/25, 25%). As determined by all investigated samples, no viruses were observed in seven different regions (7/14, 50%). Significant regional differences in the distribution patterns of the investigated viruses could be observed (Figure 2). DWV was observed with the highest frequency (60%) in Central and West Bulgaria, while in the southern regions of the country it was established with a much lower frequency (20%). The SBV virus showed a similar way of spreading. This virus was detected with the highest frequency in Central Bulgaria (40%), followed by West Bulgaria (20%). In the northern, southern, and eastern parts of the country, there was no presence of SBV. The highest rate of co-infection, i.e., simultaneous detection of DWV and SBV, was found in the central part of the country (Figure 2). These results practically showed that every studied sample from this part of the country contained both viruses. Another region of co-infection was the Sofia region, but unlike Central Bulgaria, samples with DWV presence were only observed here.

### 3.2. Molecular Phylogenetic Analysis of DWV and SBV

We were able to successfully sequence ten positive samples for DWV and three for SBV. After proper processing, fragments of 382 bp (DWV) and 417 bp (SBV) were used for phylogenetic analysis. Two positive samples for DWV and one for SBV with missing data (because of gaps in alignments) were excluded from the phylogenetic analysis. The primer sets for detection of DWV covered the part of the 3′ end of the virus genome encoding fragments of genes for nonstructural proteins chymotrypsin-like 3C protease and RdRp (nucleotide position 8566–8960 bp, from RefSeq Acc. No. NC_004830) [41]. The primer sets for detection of SBV also covered the part of the 3′ flanking region of the entire virus genome encoding segments of genes for nonstructural protein RdRp (nucleotide position 7747–8172 bp, from RefSeq Acc. No. NC_002066) [43]. The phylogenetic analysis also included viral sequences from structural, nonstructural, and full genomes, based on their availability of viral isolates from other countries.

**Figure 3:**
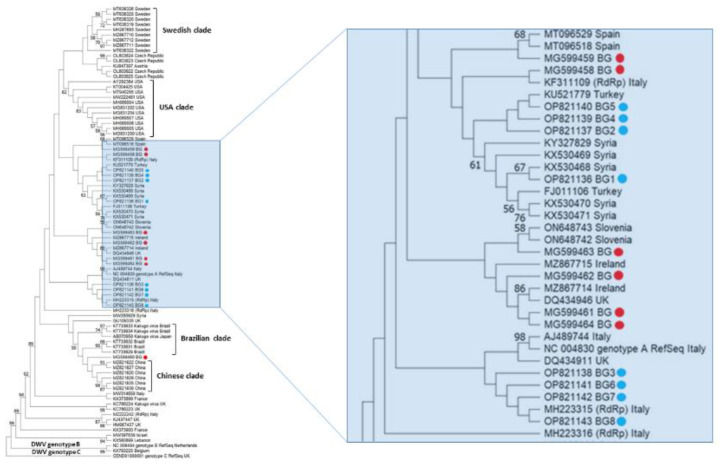
Phylogeny of deformed wing virus (DWV) isolates from Bulgaria and other countries. The unrooted phylogenetic tree was constructed based on the alignment of parts of nonstructural protein gene sequences of DWV isolates (382 bp) (nucleotide positions 8566–8960 bp according to RefSeq Acc. no. NC_004830) from Bulgaria using the neighbor-joining method under the maximum composite likelihood model. The indicated branching topology was evaluated by bootstrap resampling of the sequences 10,000 times, and nodes supported by bootstrap values > 50 are shown. Each isolate is indicated by the country of isolation and GenBank accession number. Bulgarian isolates identified by this study are shown in blue and red (the isolates from honey bees) [48].

The phylogeny of Bulgarian DWV isolates from pollen samples is shown in Figure 3. The bootstrap values of many nodes are relatively low (<50). The phylogenetic analysis of the Bulgarian DWV sequences showed that they all belonged to genotype A DWV, and the phylogenetic tree showed that it was not likely that they shared a common origin. All eight detected Bulgarian isolates were arranged in a phylogenetic tree: four of them were grouped with a common branch with an Italian isolate (No. MH223315), the other three were clustered with a Turkish one (Acc. No. KU521779), and one shared a common branch with a Syrian one (Acc. No. KX530468). This indicates that Bulgarian strains show a high degree of genetic diversity. Figure 3 clearly demonstrates that the USA clade, the Swedish clade, and the Chinese clade formed separate clusters. The monophyletic patterns of these strains show that relatively homogenous populations of DWV are present in these countries. In contrast, European DWV strains (including Bulgarian isolates) do not seem to fall within a typical European clade; instead, they can be grouped with strains from Asia and South America. This suggests that European DWV strains do not share a common origin.

**Figure 4:**
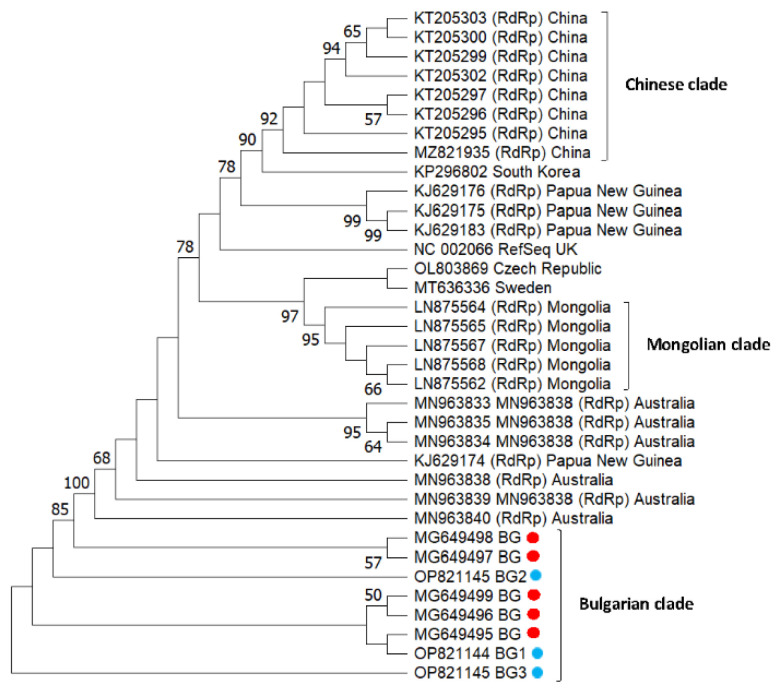
Phylogeny of sacbrood virus (SBV) isolates from Bulgaria and other countries. The unrooted phylogenetic tree was constructed based on the alignment of the part of nonstructural protein gene sequences of SBV isolates (382 bp) (nucleotide position 7747–8172 bp according to RefSeq Acc. No. NC_002066) from Bulgaria using the neighbor-joining method under the maximum composite likelihood model. The indicated branching topology was evaluated by bootstrap resampling of the sequences 10,000 times, and nodes supported by bootstrap values > 50 are shown. Each isolate is indicated by the country of isolation and GenBank accession number. Bulgarian isolates identified by this study are shown in blue and red (the isolates from honey bees) [48].

Figure 4 represents the phylogeny of Bulgarian SBV isolates from pollen samples. The bootstrap values of many nodes are relatively low (<50). All newly obtained sequences grouped together with isolates from Bulgaria (Acc. No. MG649495–MG649499) (Bulgarian clade). This shows that relatively homogenous populations of the newly obtained SBV strain with a single origin appeared to circulate among *Apis mellifera* colonies in Bulgarian apiaries. The Chinese and the Mongolian branches (with the exception of one isolate, Acc. No. KJ629183) also formed independent clades. This suggests that each country’s isolates have a common origin, and each shows geographic separation. An interesting fact is that the Australian clade also formed a separate cluster, which is most likely related to the absence of the Varroa mite (transmitting SBV) on the continent. In contrast, European isolates (except Bulgarian) did not form a separate clade; instead, they dispersed among Asian and Oceanian (Papua New Guinea) strains. These data indicate that European isolates are not geographically separated at the continent level.

## 4. Discussion

Beekeeping is more widespread in northern (200,623) and southeastern Bulgaria (168,601), where 84% of bee colonies are kept, as of 2020. The largest number of bee colonies are kept in north-central (210,830) and northeastern regions (190,417), followed by the southeastern (168,601) and northwestern regions (158,721). The population density is the highest in large cities, primarily the capital, which could facilitate virus passage from one hive to another in this region (Figure 1). An additional prerequisite for the spread of viruses could be the year-round visits to resort villages where beekeeping is practiced, located mainly in the central part of the country, which is evident from the data presented in Figure 2.

The availability of high-quality total RNA is essential for various experiments in molecular biology. The presence of complex carbohydrates and phenolic compounds in plant tissues creates problems with RNA isolation, as they bind to RNA during extraction, creating insoluble viscous material [54,55]. High RNase activity in plant tissues and their cell wall components further complicates RNA isolation [56,57]. Pollen collected using a dust trap is a valuable source of RNA, as it not subjected to enzymatic changes, and its drying at 40 ℃ does not affect RNA negatively.

This paper presents the molecular detection of the most widespread viruses in bee colonies (DWV, SBV, ABPV, CBPV, IAPV, and BQCV) in pollen samples directly taken from forager honey bees via pollen traps or bee bread. Only DWV and SBV were identified in the obtained samples. A high percentage of the investigated pollen grains and bee bread tested positive for the two viruses, especially for DWV (up to 60% in central and West Bulgaria) (Figure 1). Similarly to DWV, SBV was detected in some of the investigated pollen samples, but with lower frequencies, in two regions in the country (West and central Bulgaria). A case of co-contamination was also observed in the same regions. The obtained data correspond to human population density in these regions, which could facilitate virus passage from one hive to another. Moreover, a large number of apiaries in these regions are located at a relatively close distance from each other, which also provides an opportunity for easy virus spreading. When comparing the detected viruses in pollen grains and honey bee samples, a significant variation in the distribution of different virus species was observed. While BQCV and ABPV were identified in honey bee foragers [48], none of the samples showed the presence of these viruses in pollen grains. Conversely, the incidence of DWV was equally distributed among foragers and pollen grains. This suggests that the distribution of these viruses results from various factors, such as viral ecology in this environment or different levels of infection among pollinators when carrying the viruses with the pollen [35,37]. It is well known that honey bee viruses are not related only to the honey bee; instead, a different group of pollinators may be responsible for contamination of pollen [58,59]. The fact that DWV was the most prevalent virus in the wild pollinators [60,61] presupposes the increased detection of DWV in pollen grains. Moreover, it has been observed that detection of DWV and SBV in pollen loads is not directly related to infecting foragers (carrying pollen), thus suggesting that some foragers may have been bringing the virus from outside and thereby directly implicating pollen as a source of virus infection for healthy colonies [62,63]. Further evidence that pollinators directly transfer viruses from one plant to another is the fact that plants have been found to be “viral hot spots” by researchers in the field [64]. This fact suggests that pollinators may become infected with a new virus introduced to the environment by another pollinator, mediated by plant pollen.

We performed a phylogenetic analysis to search for possible differences among honey bee viruses collected from pollen as well as from foragers. A comparison of viral sequences (DWV and SBV) from honey bee foragers and pollen grains revealed different relationships which are directly dependent on the virus species. Neighbor-joining unrooted phylogenetic trees were constructed for DWV (Figure 3) and SBV (Figure 4), based on parts of genes encoding nonstructural proteins. For DWV, no sequences obtained from pollen grains or bee bread shared common branches with the virus isolated from the honey bee. This suggests that the virus found in pollen was most likely previously deposited on the flowers by other pollinators infected with the virus. Another possible explanation for the different grouping of DWV may be associated with the *Varroa destructor* mite. In our previous study, the presence of the Varroa mite was not checked for each colony at the time of sampling [48]. Therefore, the DWV found in honey bees may have been directly injected from the *Varroa destructor*. When DWV is vectored by the Varroa mite, it is possible that different strains enter honey bees. Further proof of this has been provided by studies showing that DWV infection can occur with several variants cocirculating in the same apiary, colony, or individual honey bee host [65,66,67]. Regarding SBV phylogeny, cladograms show a different scenario. Despite the small number of isolates (only three), Figure 4 illustrates that two strains of the virus found in pollen grains show a different phylogeny than the bee virus isolates. Only one isolate (Acc. No. OP821144) was clustered together with a honey bee isolate (MG649495), which suggests that the same virus strain was detected in honey bees and pollen.

## 5. Conclusions

This study has reported, for the first time, the molecular detection of DWV and SBV in pollen grains and bee bread in the Balkan region. This strategy represents a noninvasive approach that allows for monitoring of viral diseases in honey bees at any time at a regional and a national level. Since the majority of viral diseases occur without clear clinical symptoms, their detection in bee products represents a reliable strategy for their prevention. The research carried out herein is important for beekeeping practice, as it shows the possibility of infecting healthy bee colonies when replenishing the food supplies of the infected colonies. Therefore, before taking these steps, it is necessary to examine the health status of the bee colony from which the food sources will be provided. The dynamics of viral transmission by pollen must be further investigated to determine whether pollen has a greater role than simply as a physical source of these viruses.

## Figures and Tables

**Table 1:** Primers used for RT-PCR detection of honey bee-associated viruses.

Primer	Sequence (5′-3′)	Length (bp) ^1^	Reference
DWV-F	TTTGCAAGATGCTGTATGTGG	395	[40]
DWV-R	GTCGTGCAGCTCGATAGGAT		
SBV-F	GGATGAAAGGAAATTACCAG	426	[42]
SBV-R	CCACTAGGTGATCCACACT		
ABPV-F	TGAGAACACCTGTAATGTGG	452	[42]
ABPV-R	ACCAGAGGGTTGACTGTGTG		
BQCV-F	GGACGAAAGGAAGCCTAAAC	424	[42]
BQCV-R	ACTAGGAAGAGACTTGCACC		
CBPV-F	AGTTGTCATGGTTAACAGGATACGAG	455	[44]
CBPV-R	TCTAATCTTAGCACGAAAGCCGAG		
KBV-F	GATGAACGTCGACCTATTGA	393	[47]
KBV-R	TGTGGGTTGGCTATGAGTCA		

^1^ Length of cDNA fragment amplified.

## Data Availability

All data are available upon request.
